# Serosurvey of *Toxoplasma gondii* and *Toxocara* spp. co-infection in pregnant women in low-income areas of Brazil

**DOI:** 10.3389/fpubh.2024.1340434

**Published:** 2024-01-25

**Authors:** Edlayne Larissa Gretter Machado Pereira, Isabella Braghin Ferreira, Roberta Brinholi Victorino, Susana Angélica Zevallos Lescano, Rogério Giuffrida, Louise Bach Kmetiuk, Alexander Welker Biondo, Vamilton Alvares Santarém

**Affiliations:** ^1^Graduate College in Animal Sciences, University of Western São Paulo (UNOESTE), São Paulo, Brazil; ^2^Medical School, University of Western São Paulo (UNOESTE), São Paulo, Brazil; ^3^Institute of Tropical Medicine of São Paulo, University of São Paulo, São Paulo, Brazil; ^4^Zoonoses Surveillance Unit, Municipal Secretary of Health, Curitiba, Brazil; ^5^Graduate College of Cell and Molecular Biology, Federal University of Paraná (UFPR), Curitiba, Brazil

**Keywords:** epidemiology, pregnancy, toxocariasis, toxoplasmosis, zoonoses

## Abstract

**Introduction:**

Despite human toxoplasmosis and toxocariasis having been listed among the top six most neglected parasitic zoonoses worldwide, presenting similar associated risk factors and transmission routes, few studies have been conducted in pregnant women and the consequences of concurrent infection remain to be fully established. Accordingly, the present study has serologically assessed the co-infection and associated risk factors for *Toxoplasma gondii* and *Toxocara* spp. in pregnant women, assisted by the public Unified National Health System (SUS) in southeastern Brazil.

**Materials and Methods:**

Blood samples were collected and tested for IgG antibodies against *Toxoplasma gondii* by chemiluminescence immunoassay and against *Toxocara* spp. by enzyme-linked immunosorbent assay (ELISA). An epidemiological questionnaire was applied to gather socioeconomic information to assess the risk factors associated with seropositivity to toxocariasis/toxoplasmosis by univariate analysis followed by logistic regression.

**Results:**

Overall, seropositivity was 69/280 (24.6, 95% CI: 19.96–30.01) for *T. gondii* and 56/280 (20.0, 95% CI: 15.73–25.08) for *Toxocara* spp. Co-infection was observed in 25/280 (8.9, 95% CI: 6.12–12.85) pregnant women, with increased odds (OR: 3.3, CI 95%: 1.77–6.14, *p* = 0.0002). Logistic regression revealed that a higher educational level (high school or college) significantly reduced the likelihood of co-infection seropositivity, owning cats increased the odds of toxocariasis, and older pregnant women presented significantly higher *T. gondii* seropositivity.

**Conclusion:**

Co-infection herein highlights the importance of educational programs in the prevention of toxocariasis and toxoplasmosis in pregnant women and other high-risk populations.

## 1 Introduction

Toxoplasmosis and toxocariasis, caused by the *Toxoplasma gondii* protozoan and *Toxocara* spp. nematodes, respectively, have been important parasitic zoonoses of public health concern (1). Both diseases have been highly associated with low socioeconomic conditions (2, 3), with a global overall prevalence of 19% for toxocariasis (4) and 36% for toxoplasmosis (5). Pregnant women have also been highly affected by both infections, with global seroprevalence of 1.9 and 32.9% for anti-*T. gondii* IgM and IgG, respectively (6), and toxocariasis prevalence of 6.4% worldwide, 7.4% in Brasília, central-western Brazil (7), 9.2% in the Shandong Province, eastern China (8), 14.5% in Caribbean countries (9), 17.2% in Athens and nearby Piraeus, Greece (10), and 21.2% in the Ilam Province, western Iran (11).

Although toxoplasmosis may be asymptomatic in approximately 50% of healthy immunocompetent adults, severe infection with devastating sequelae has been reported in immunocompromised individuals and neonates (12). Thus, pregnant women have been considered the most vulnerable group in need of healthcare attention to prevent and monitor toxoplasmosis infection (5). In addition, transplacental transmission of *T. gondii* may lead to fetal miscarriage or stillbirth, severe disease in living infants (3), and permanent injuries including neurological damage and blindness (13).

Toxocariasis agents have also been asymptomatic in most human infections, but serious health consequences (14) may occur, including hepatic (15), pulmonary (16), cardiac (17), and urinary (18) lesions in the visceral form, ocular impairment or vision loss (19, 20), and neurological disorders (21, 22). Although rare, congenital transmission of *Toxocara* spp. was reported to cause ocular lesions in a premature child in Argentina (23) and strabismus in a 5-week-old infant in the USA (24). Vertical transmission has been experimentally reproduced in a murine model for both *Toxocara canis* (25) and *Toxocara cati* (26).

Toxoplasmosis and toxocariasis have shared several associated risk factors, particularly due to low socioeconomic aspects (27). In addition to similar human organ targeting, both pathogens have been directly transmitted to the human hosts through the intake of raw or undercooked meat or viscera from intermediate hosts of *T. gondii* or paratenic hosts of *Toxocara* spp. (28) and through the ingestion of *T. gondii* sporulated oocysts (29) or infective *Toxocara* spp. eggs from soil, vegetables, and water (30, 31).

Despite the similarities of infection, transmission, and associated risks of toxoplasmosis and toxocariasis for pregnant women, the prevalence and impact of co-infection remain to be fully established. Accordingly, the present study has simultaneously assessed infection by *T. gondii* and *Toxocara* spp. and the risk factors associated with seropositivity in pregnant women assisted by the Public Health System in southeastern Brazil.

## 2 Materials and methods

### 2.1 Ethics statement

The present study was approved for research with human beings by the Ethics Committee at the University of Western São Paulo (UNOESTE), corroborated by the Brazilian National Health Council (protocol number 52817021.0.0000.5515).

For blood and data collection including socioepidemiological and socioeconomic information, individuals were informed about the survey and personal confidentiality. Participants formalized the authorization by signing a Free and Informed Consent Term (FICT), in compliance with the Brazilian National Health Council.

### 2.2 Study design

The present study was a cross-sectional serosurvey of anti-*Toxocara* spp. and anti-*T. gondii* antibodies (IgG) and the associated risk factors in pregnant women, assisted by the public Unified National Health System in southeastern Brazil.

### 2.3 Timeline and study area

The study was conducted from March to October 2022 in the city of Presidente Prudente, São Paulo state, southeastern Brazil, ranked 136^th^ nationwide (top 2.4%) for population, with approximately 225,000 habitants, 146^th^ (top 2.6%) for Gross Domestic Product - GDP, and 25^th^ (top 0.4%) for Human Development Index - HDI (0.806), out of 5,570 Brazilian cities (32). Nonetheless, approximately 30% of inhabitants lived on half the minimum monthly wage (approximately US$ 245) at the time.

### 2.4 Sample size

The parameters to estimate the sample size were calculated based on a previous prevalence study of toxoplasmosis and toxocariasis in 280 pregnant women from southeastern Brazil, with the assumption of an expected co-infection rate of 3.2%, a 95% confidence interval, and 10% losses (33).

### 2.5 Blood sample collection and epidemiological information

All women who participated in the present survey voluntarily answered an epidemiological questionnaire and had blood samples collected to assess the presence of anti-*T. gondii* and anti-*Toxocara* spp. antibodies. Official consent forms were signed by adult pregnant women and the legal guardians of underage adolescents younger than 18 years old, as required by current Brazilian laws.

Blood sampling was performed by certified nurses from two prenatal reference centers at the City Secretary of Health of Presidente Prudente. A total of 10 mL was collected by venipuncture, placed into a tube with serum separator gel, and centrifuged at 800 × *g* for 5 min; the serum was then separated and kept at −20°C until laboratory processing.

The applied questionnaire gathered socioepidemiological and socioeconomic information to assess the associated risk factors for toxocariasis and toxoplasmosis (Table 1).

**Table 1:** Gathered information for assessing the potential exposure and associated risk factors for toxocariasis and toxoplasmosis.

Topics	Information
Socioeconomic characteristics	Age; gestational stage; educational level; family income (minimum wages per month); presence of sewage system at the household
Domestic animals	Owning a dog or cat
Practices	Intake of filtered water; soil contact; nail biting; allotriophagy; ingestion of raw or undercooked meat; vegetable sanitation

Subjects were excluded from the present study if they failed to present a medical request for prenatal blood examination or refused to voluntarily sign the FICT provided by the researchers.

### 2.6 Serological tests

Detection of anti-*Toxoplasma gondii* antibodies (IgG) was performed during prenatal care by chemiluminescence microparticle immunoassay using a commercial kit test (Ortho-Clinical Diagnostics, Illkirch-Graffenstaden, France), performed in two city reference centers for the prenatal diagnosis of toxoplasmosis. Anti-*Toxocara* spp. antibodies (IgG) were detected using an in-house indirect enzyme-linked immunosorbent assay (ELISA) through antigen excretion and secretion (TES) of *Toxocara canis*, following a protocol of pre-adsorption with *Ascaris suum* antigen (34) of each tested sample to minimize the potential cross-reactivity by exposure to *Ascaris* spp. A serum previously shown to be non-reactive (negative control) and a known reactive serum (positive control) were tested along with research samples on each plate. Absorbance was read at 492 nm, and antibody levels were expressed as reactivity indexes (RI), which were calculated as the ratio between the absorbance values of each sample and the cut-off value of the ELISA test. Testing of anti-*Toxocara* spp. antibodies was carried out at the Institute of Tropical Medicine of São Paulo, São Paulo state, Brazil.

### 2.7 Statistical analysis

All the statistical analyses were performed using R software (35). Seropositivity to toxocariasis and toxoplasmosis were independently compared using Pearson’s chi-square test. To access risk factors related to seropositivity, outcome data were initially categorized (variables shown in Table 1) and submitted to the univariate analysis (Pearson Chi-Squared Test or Fisher’s exact test). Variables presenting statistical significance lower than 0.20 in the univariate model were included in multivariate analyses (logistical regression) to assess the contribution of the risk/protective factors studied to the likelihood of seropositivity. To improve the final model, the predictor variables were tested for collinearity and the presence of influential values. From the regression coefficients for each predictor variable, odds ratio values were estimated per point and with a 95% confidence interval. The best-fitting model was considered the one that included significantly associated variables (*p*-value < 0.05) and minimized the Akaike Information Criterion (AIC) value. A significant level of 5% was adopted for all statistical tests.

## 3 Results

### 3.1 Characteristics of the studied population

The ages of pregnant women in the present study ranged from 15 to 43 years (median = 26); most were adults (267/280, 95.4%) and 13 were underage individuals (less than 18 years old). The majority of women (177/280, 63.0%) reported a family monthly income of up to 2 minimum wages, while more than half of adult women (145/267, 54.3%) declared having a work occupation. Pregnant women mostly (194/280, 69.3%) self-declared a history of previous pregnancy.

### 3.2 Prevalence and concurrent infection to *Toxoplasma gondii* and *Toxocara* spp.

Overall seropositivity (IgG) was observed in 69/280 (24.6, 95% CI: 19.96–30.01) women to *T. gondii*, and in 56/280 (20.0, 95% CI: 15.73–25.08) women to *Toxocara* spp., resulting in a 1.23 ratio. Concomitant seropositivity was verified in 25/280 (8.9, 95% CI: 6.12–12.85) individuals, with a high statistically significant association between *Toxocara* spp. and *T. gondii* seropositivity (OR: 3.3, CI 95%: 1.77–6.14, *p* = 0.0002).

### 3.3 Risk factors for *Toxocara* spp. infection and for *Toxoplasma gondii* infection

Risk factors associated with seropositivity to anti-*Toxocara* spp. antibodies (IgG) were gathered and analyzed (Table 2). Logistic regression (multivariate analysis) revealed that having a high school (OR: 0.26, *p* < 0.001) or college (OR: 0.23, *p* = 0.005) degree and the presence of a sewage system at home (OR: 0.08, *p* = 0.036) were protective factors, while owning cats was a risk factor and increased the odds (OR: 2.3, *p* = 0.033) of toxocariasis in pregnant women.

**Table 2:** Association of the presence of anti-*Toxocara* spp. antibodies (IgG) with characteristics of pregnant women (*N* = 280) assisted by the Public Health System in southeastern Brazil, by univariate and logistic multivariate regression analysis.

	Positive no. (%)	Negative no. (%)	Univariate analysis		Multivariate analysis	
Variables	56 (20.0)	224 (80.0)	OR (95% CI)	*p-*overall	OR (95% CI)	*p*-value
**Age (years old)**				0.811		
15–21	9 (16.1)	49 (21.9)	1.0 [Reference]			
22–25	14 (25.0)	52 (23.2)	1.45 (0.58–3.82)			
26–30	17 (30.3)	61 (27.2)	1.50 (0.62–3.84)			
31–43	16 (28.6)	62 (27.7)	1.39 (0.57–3.58)			
**Educational level**				<0.001		
Elementary	22 (39.3)	31 (13.9)	1.0 [Reference]		1.0 [Reference]	
High school	27 (48.2)	145 (65.0)	0.26 (0.13–0.53)		0.26 (0.12–0.54)	<0.001
College	7 (12.5)	47 (21.1)	0.22 (0.08–0.55)		0.23 (0.08–0.61)	0.005
**Income (mw/month)**				0.515		
Up to 1.9	39 (69.7)	147 (66.2)	1.0 [Reference]			
2 up to 3.9	13 (23.2)	47 (21.2)	1.05 (0.50–2.10)			
4.0 or more	4 (7.1)	28 (12.6)	0.56 (0.15–1.54)			
**Filtered water**				0.624		
No	38 (67.9)	141 (63.2)	1.0 [Reference]			
Yes	18 (32.1)	82 (36.8)	0.82 (0.43–1.51)			
**Sewage system at home**				0.025		
No	3 (5.5)	1 (0.4)	1.0 [Reference]		1.0 [Reference]	
Yes	52 (94.5)	223 (99.6)	0.09 (0.00–0.75)		0.08 (0.00–0.70)	0.036
**Soil contact**				0.135		
No	31 (56.4)	148 (68.2)	1.0 [Reference]			
Yes	24 (43.6)	69 (31.8)	1.66 (0.90–3.04)			
**Owning dogs**				0.593		
No	17 (30.4)	79 (35.3)	1.0 [Reference]			
Yes	39 (69.6)	145 (64.7)	1.24 (0.67–2.40)			
**Owning cats**				0.012		
No	39 (69.6)	190 (85.2)	1.0 [Reference]		1.0 [Reference]	
Yes	17 (30.4)	33 (14.8)	2.51 (1.25–4.93)		2.27 (1.05–4.78)	0.033
**Nail biting**				0.878		
No	33 (58.9)	137 (61.2)	1.0 [Reference]			
Yes	23 (41.1)	87 (38.8)	1.10 (0.60–1.99)			
**Allotriophagy**				0.562		
No	36 (64.3)	132 (58.9)	1.0 [Reference]			
Yes	20 (35.7)	92 (41.1)	0.80 (0.43–1.46)			
**Ingestion of raw/undercooked meat**				0.613		
No	46 (82.1)	173 (77.9)	1.0 [Reference]			
Yes	10 (17.9)	49 (22.1)	0.78 (0.35–1.60)			
**Vegetable sanitation**				0.111		
Water	39 (72.2)	142 (66.4)	1.0 [Reference]		1.0 [Reference]	
Sodium hypochlorite	9 (16.7)	23 (10.7)	1.43 (0.58–3.29)		1.38 (0.51–3.42)	0.507
Vinegar	6 (11.1)	49 (22.9)	0.46 (0.16–1.08)		0.50 (0.17–1.21)	0.150
**Gestational stage (trimester)**				0.817		
First	27 (48.2)	102 (45.6)	1.0 [Reference]			
Second	12 (21.4)	44 (19.6)	1.04 (0.46–2.20)			
Third	17 (30.4)	78 (34.8)	0.83 (0.41–1.62)			

mw, minimum wage by family per month.

The final multivariate model for *T. gondii* seropositivity was calculated and presented (Table 3). Having a high school (OR: 0.44, *p* < 0.016) or college (OR: 0.17, *p* < 0.001) degree was a statistically significant protective factor for toxoplasmosis. In addition, the odds of *T. gondii* seropositivity were directly proportional to the age of pregnant women, considering the ages of 15 to 21 as a reference.

**Table 3:** Association between the presence of anti-*Toxoplasma gondii* antibodies (IgG) and characteristics of pregnant women (*N* = 280) assisted by the Public Health System in southeastern Brazil, by univariate and logistic multivariate regression analysis.

	Positive no. (%)	Negative no. (%)	Univariate analysis		Multivariate analysis	
Variables	69 (24.6)	211 (75.4)	OR (95% CI)	*p-*overall	OR (95% CI)	*p*-value
**Age (years old)**				0.001		
15–21	6 (8.7)	52 (24.6)	1.0 [Reference]		1.0 [Reference]	
22–25	10 (14.5)	56 (26.5)	1.53 (0.52–4.87)		1.53 (0.52–4.85)	0.453
26–30	25 (36.2)	53 (25.1)	3.98 (1.58–11.6)		5.13 (1.97–15.26)	0.001
31–43	28 (40.6)	50 (23.8)	4.72 (1.89–13.7)		5.59 (2.13–16.78)	<0.001
**Educational Level**				0.021		
Elementary	20 (29.0)	33 (15.7)	1.0 [Reference]		1.0 [Reference]	
High School	41 (59.4)	131 (62.4)	0.52 (0.27–1.01)		0.44 (0.20–0.85)	0.016
College	8 (11.6)	46 (21.9)	0.29 (0.11–0.73)		0.17 (0.06–0.44)	<0.001
**Income (mw/month)**				0.726		
Up to 1.9	47 (69.1)	139 (66.2)	1.0 [Reference]			
2 up to 3.9	15 (22.1)	45 (21.4)	0.99 (0.49–1.92)			
4.0 or more	6 (8.8)	26 (12.4)	0.70 (0.24–1.71)			
**Filtered water**				0.519		
No	47 (68.1)	132 (62.9)	1.0 [Reference]			
Yes	22 (31.9)	78 (37.1)	0.80 (0.44–1.41)			
**Sewage system at home**				1.0		
No	1 (1.5)	3 (1.4)	1.0 [Reference]			
Yes	67 (98.5)	208 (98.6)	0.89 (0.10–25.8)			
**Soil contact**				0.442		
No	41 (61.2)	138 (67.3)	1.0 [Reference]			
Yes	26 (38.8)	67 (32.7)	1.31 (0.73–2.31)			
**Owning cats**				0.257		
No	53 (76.8)	176 (83.8)	1.0 [Reference]			
Yes	16 (23.2)	34 (16.2)	1.57 (0.78–3.03)			
**Nail biting**				0.111		
No	48 (69.6)	122 (57.8)	1.0 [Reference]		1.0 [Reference]	
Yes	21 (30.4)	89 (42.2)	0.60 (0.33–1.07)		0.71 (0.37–1.33)	0.292
**Allotriophagy**				0.756		
No	43 (62.3)	125 (59.2)	1.0 [Reference]			
Yes	26 (37.7)	86 (40.8)	0.88 (0.50–1.54)			
**Ingestion of raw/undercooked meat**				0.715		
No	52 (76.5)	167 (79.5)	1.0 [Reference]			
Yes	16 (23.5)	43 (20.5)	1.20 (0.61–2.28)			
**Vegetable sanitation**				0.336		
Water	41 (61.2)	140 (69.7)	1.0 [Reference]			
Sodium hypochlorite	11 (16.4)	21 (10.4)	1.79 (0.77–3.99)			
Vinegar	15 (22.4)	40 (19.9)	1.28 (0.63–2.53)			
**Gestational stage (trimester)**				0.248		
First	35 (50.7)	94 (44.5)	1.0 [Reference]			
Second	9 (13.1)	47 (22.3)	0.52 (0.22–1.14)			
Third	25 (36.2)	70 (33.2)	0.96 (0.52–1.75)			

mw, minimum wage by family per month.

Previous miscarriage was not associated with a positive serological result to toxocariasis (OR: 0.6, CI 95%: 0.287–1.258, *p* = 0.238) nor toxoplasmosis (OR: 0.7, CI 95%: 0.376–1.422, *p* = 0.449). The ROC curve presenting the model’s accuracy was constructed (Supplementary Figure S1), with 70.3% for toxocariasis and 71.0% for toxoplasmosis, which were considered fair (36).

## 4 Discussion

Seroprevalence of *Toxocara* spp. and *Toxoplasma gondii* was observed in the present study, with a significant concurrent infection in pregnant women living in low-income areas of southeastern Brazil.

The 20.0% (56/280) seropositivity to toxocariasis herein was higher than the 6.4% found in southern Brazil (33) and the 7.2% observed in central-western Brazil (7), both in pregnant women assisted by the Public Health System (SUS), but similar to the 20.7% observed in a different group of pregnant women in Presidente Prudente (37), the municipality of the present study. Although all these low-income populations were assisted by SUS and presented disadvantaged socioeconomic characteristics, differences in seroprevalence may reflect local infrastructure, habits, and hygiene. Nonetheless, the results herein are similar to the overall global 19.0% toxocariasis seropositivity, which has been associated with lower income levels (4).

The 24.6% (69/280) seropositivity to *T. gondii* herein is lower than that of other recent Brazilian studies (last 10 years) involving pregnant women attended by SUS, ranging from 51.7% in southern Brazil (38) to 68.4% in northern Brazil (39). Although such variation could be related to the technique used for IgG detection, eating habits and water sources may play a role in transmission, as already reported (13). In addition, such seropositivity divergence between the two pregnant populations may be influenced by the socio-economic and climatic differences. As the northern Tocantins state presents higher rainfall and temperature, favoring the disease cycle, and the southern Paraná shows a very similar climate to the study setting herein, the socio-economic disparities between these two regions may be the main reason for seropositivity differences.

In the present study, a statistically significant concurrent 8.9% seropositivity (25/280, OR: 3.3) to anti-*T. gondii* and anti-*Toxocara* spp. differed from previous pregnant women serosurveys, with 7.9% (22/280) in southern Brazil (33), 13.2% (31/235) in China (40), and 9.5% (36/378) in western Iran (41). Co-infection has been shown in pregnant women, as IgG antibodies against *T. gondii* posed a risk (OR: 2.2, 95% CI: 1.7–2.9) of *Toxocara* spp. seropositivity in a general population survey of 13,509 individuals between 2011 and 2014 (42). Co-infection has also been reported in children, ranging from 3.2% (17/544) (43) to 27.4% (113/412) (27) in southern Brazil.

Herein, the ratio between toxoplasmosis/toxocariasis seroprevalence (1.23) was higher than 1. It was very close to the 1.17 observed in China (40) and lower than the 4.2 in western Iran (41). This finding possibly indicates increased exposure of pregnant women to *T. gondii* herein. In Iran, seropositivity in pregnancy was associated with contact with cats (41). In the present study, aging was associated with increased odds of *T. gondii* seropositivity, as shown in older pregnant women in northeastern (44, 45), southern (46, 47), northern (48), and central-western Brazil (49), Iran (50), and Saudi Arabia (51). This phenomenon has been attributed to lifelong antibody persistence and detection (52), but other causes have been indicated such as prolonged exposure to etiological agents, transmission routes, and lack of public awareness about prevention (50). Although increased odds of *Toxocara* spp. seropositivity has also been related to antibody persistence following exposure (53), no association between age and seropositivity was observed herein. This outcome differs from a previous study in the same area, in which adolescents were 2.6-fold more likely to be seropositive for toxocariasis than adult pregnant women (37), showing soil contact as an associated risk factor for toxocariasis among pregnant adolescents, but not for pregnant adults.

In Brazil, previous studies have reported risk factors associated with *T. gondii* infection during pregnancy, such as previous contact with cats in northern (54), northeastern (45, 55), and southern states (46, 56); socioeconomical vulnerability (55, 57), washing vegetables with untreated water (57), dog ownership (57), and homemade water ice consumption in northeastern states (57); living in urban area (48), frequent consumption of vegetables (58), and meat handling in northern states (54); living in rural areas in southern states (38, 47, 59, 60); gardening activities, contact with soil (48, 61), and raw milk consumption in southern and northern states (56, 58); consumption of chicken and meat in northeastern and northern states (57, 58). However, few studies involving co-infection with both parasites in pregnant women have been conducted, limiting the discussion of comparative risk factors. Thus, further retrospective and review studies are necessary to characterize the ratio of co-infection in different pregnant populations.

The similar transmission route of *T. gondii* and *Toxocara* spp., including consumption of raw or undercooked meat and the ingestion of embryonated eggs of *Toxocara* spp. and oocysts of *T. gondii*, may explain the co-infection in pregnant women observed in southern Brazil (33). Herein, logistic regression revealed that having a higher educational level was a protective factor against toxoplasmosis and toxocariasis co-seropositivity, corroborating with other serosurveys for *T. gondii* in pregnant women in Brazil (38, 55, 62) and other countries, such as Benin, Africa (63). Pregnant women with a higher educational level were associated with having toxoplasmosis-related knowledge (62), which may increase their awareness and understanding of the importance of hygiene habits to prevent diseases, including toxoplasmosis (64). Although the presence of a sewage system at home herein was also found to be a protective factor for toxocariasis but not for toxoplasmosis, having no public sewer service was previously associated with an increased risk of toxoplasmosis in southern Brazil (65).

In addition, owning cats increased the odds (OR: 2.3) of toxocariasis seropositivity herein, while no influence had previously been reported in that area (37). Despite the pregnant population of both studies being similar, the divergent outcome between studies may have been influenced by the higher number of pregnant women who declared their ownership of cats herein (n = 50) and previously (n = 38), which may have made it difficult to find statistically significant differences due to the low number of positive individuals. Likewise, owning cats has been reported as a risk factor for toxoplasmosis in some (66–69) but not all (27, 45, 70) serosurveys worldwide. Although seropositivity to toxoplasmosis herein was not influenced by the presence of cats in the household, increased odds of *Toxocara* spp. seropositivity herein due to cat contact should be considered as a potential disease risk when keeping cats at home.

Pregnant women may be directly infected by *T. gondii* and *Toxocara* spp. through the consumption of raw or undercooked meat or viscera (28), with high odds (OR: 5.7) of toxoplasmosis observed in pregnant women in northeastern Brazil (67). Although no study has found such a risk for toxocariasis and approximately one-fifth of the pregnant women herein declared the habit of consuming undercooked or raw meat, no association with toxocariasis or toxoplasmosis seropositivity was found. As previously stated, Presidente Prudente has been ranked in the top 2.4% of cities for population nationwide, the top 2.6% for Gross Domestic Product, and the top 0.4% for Human Development Index, with low commercial meat disease risk, mostly slaughtered, traded, and handled under rigorous state and federal sanitary inspections (37).

Pregnancy may be the highest risk time to be infected with *T. gondii*, as transplacental infection may lead to a wide variety of manifestations including miscarriage, stillbirth, and severe disease in living infants, despite mostly children being asymptomatic at birth (3). In addition, transplacental toxocariasis has been reported in one premature (23) and one 5-week-old (24) baby with ocular lesions. However, no study in pregnant women has found an association between miscarriage history and toxocariasis seropositivity in Brazil (7, 37, 71), including the present study.

A limitation of the present study is that no differentiation between acute and chronic phases was made, as no IgM or antibody avidity tests were performed for either pathogen. Nonetheless, infant mortality associated with congenital toxoplasmosis has been a persistent public health problem in Brazil (72). A surveillance protocol for gestational toxoplasmosis has been recently established by the Brazilian Minister of Health, including systematic serodiagnosis, compulsory notification, and educational and preventive activities coordinated by the Unified Health System (73). Given that serological tests may result in the overestimation of prevalence via false positive results, a second test should be recommended for further confirmation in epidemiological investigations of toxoplasmosis. Accordingly, a meta-analysis revealed that a combination of tests may improve the sensitivity and provide improved accuracy (74). Finally, as knowledge of risk factors for toxoplasmosis and toxocariasis may be helpful during early pregnancy (44), such educational and preventive activities should be taken into consideration as public health policies.

## 5 Conclusion

In conclusion, significant co-infection observed herein reinforces the importance of educational programs aimed at the prevention of toxocariasis and toxoplasmosis, particularly in pregnant women at high risk of exposure.

## Data availability statement

The original contributions presented in the study are included in the article/Supplementary material, further inquiries can be directed to the corresponding author.

## Ethics statement

The present study was approved for research with human beings by the Ethics Committee at the University of Western São Paulo (UNOESTE), corroborated by the Brazilian National Health Council (protocol number 52817021.0.0000.5515). The studies were conducted in accordance with the local legislation and institutional requirements. Written informed consent for participation in this study was provided by the participants or/and their legal guardians/next of kin.

## Author contributions

EP: Conceptualization, Data curation, Formal analysis, Funding acquisition, Investigation, Validation, Visualization, Writing – original draft, Writing – review & editing. IF: Conceptualization, Data curation, Formal analysis, Investigation, Methodology, Writing – original draft, Writing – review & editing. RV: Data curation, Investigation, Writing – original draft, Writing – review & editing. SL: Formal analysis, Investigation, Methodology, Writing – original draft, Writing – review & editing. RG: Data curation, Formal analysis, Investigation, Software, Validation, Writing – original draft, Writing – review & editing. LK: Investigation, Writing – review & editing. AB: Funding acquisition, Investigation, Writing – review & editing. VS: Conceptualization, Data curation, Formal analysis, Funding acquisition, Investigation, Methodology, Project administration, Resources, Supervision, Validation, Visualization, Writing – original draft, Writing – review & editing.
