# Supplementary material for: Serosurvey of Toxoplasma gondii and Toxocara spp. co-infection in pregnant women in low-income areas of Brazil
Source: Front Public Health. 2024 Jan 25;12:1340434. doi: 10.3389/fpubh.2024.1340434 (PMC10850292; doi:10.3389/fpubh.2024.1340434)
Supplement: Supplementary file 1 [file Data_Sheet_1.docx]

Supplementary Material

# Supplementary Data


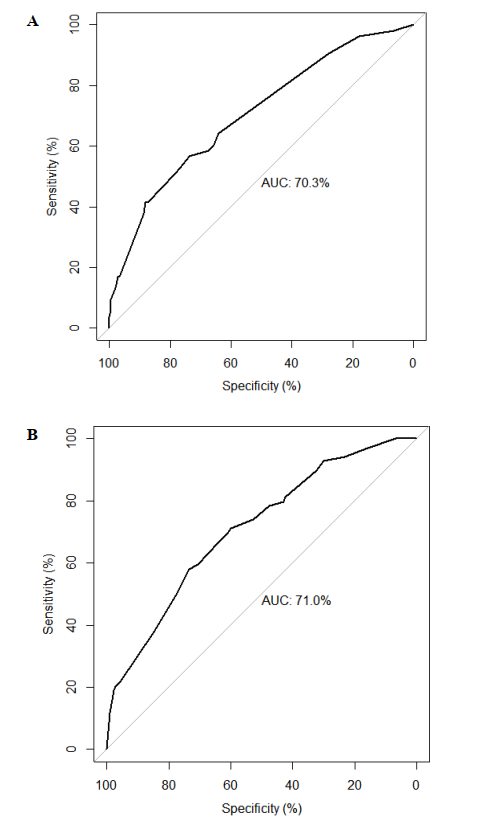


**Supplementary Figure 1.** Receiver operating characteristic (ROC) curve assessing the accuracy of the multivariate logistic regression model for predicting seropositivity for A) anti-*Toxocara* spp. [area under the curve (AUC): 0.703; 95% CI: 0.626-0.782] and B) *Toxoplasma gondii* (AUC: 0.710; 95% CI: 0.641-0.780) antibodies in 280 pregnant women in southeastern Brazil.
